# MSeg-Net: A Melanoma Mole Segmentation Network Using CornerNet and Fuzzy *K*-Means Clustering

**DOI:** 10.1155/2022/7502504

**Published:** 2022-10-14

**Authors:** Marriam Nawaz, Tahira Nazir, Muhammad Attique Khan, Majed Alhaisoni, Jung-Yeon Kim, Yunyoung Nam

**Affiliations:** ^1^Department of Software Engineering, University of Engineering and Technology Taxila, 47050, Pakistan; ^2^Department of Computer Science, University of Engineering and Technology Taxila, 47050, Pakistan; ^3^Department of Computing, Riphah International University, Islamabad, Pakistan; ^4^Department of Computer Science, HITEC University, Taxila, Pakistan; ^5^Computer Sciences Department, College of Computer and Information Sciences, Princess Nourah Bint Abdulrahman University, Riyadh 11671, Saudi Arabia; ^6^Department of ICT Convergence, Soonchunhyang University, Asan 31538, Republic of Korea

## Abstract

Melanoma is a dangerous form of skin cancer that results in the demise of patients at the developed stage. Researchers have attempted to develop automated systems for the timely recognition of this deadly disease. However, reliable and precise identification of melanoma moles is a tedious and complex activity as there exist huge differences in the mass, structure, and color of the skin lesions. Additionally, the incidence of noise, blurring, and chrominance changes in the suspected images further enhance the complexity of the detection procedure. In the proposed work, we try to overcome the limitations of the existing work by presenting a deep learning (DL) model. Descriptively, after accomplishing the preprocessing task, we have utilized an object detection approach named CornerNet model to detect melanoma lesions. Then the localized moles are passed as input to the fuzzy *K*-means (FLM) clustering approach to perform the segmentation task. To assess the segmentation power of the proposed approach, two standard databases named ISIC-2017 and ISIC-2018 are employed. Extensive experimentation has been conducted to demonstrate the robustness of the proposed approach through both numeric and pictorial results. The proposed approach is capable of detecting and segmenting the moles of arbitrary shapes and orientations. Furthermore, the presented work can tackle the presence of noise, blurring, and brightness variations as well. We have attained the segmentation accuracy values of 99.32% and 99.63% over the ISIC-2017 and ISIC-2018 databases correspondingly which clearly depicts the effectiveness of our model for the melanoma mole segmentation.

## 1. Introduction

The abnormal growth of the skin cells results in cancer inside the human body which is broadly categorized into three types namely squamous cell carcinoma, melanoma, and basal, respectively [1]. Among all three types, melanoma is designated as the most fatal type of skin cancer that develops inside the skin cells namely melanocytes. In a recent study published in [2], it is found that only in the US, approximately 10 thousand victims are dying annually due to this dangerous disease. The unnecessary expansion of the skin cells generates lesions in the human body that differ in structure, appearance, and mass. The irregular lesion is about 6 mm in size and contains a rare appearance usually in red, brown, pink, or black color which requires an urgent inspection by the dermatologist. Melanoma is further distributed into two categories named benign and malignant. The first category of melanoma known as benign is the less fatal type of skin cancer and is easily curable, whereas malignant is the advanced stage of skin cancer that may cause the victim death if not detected timely. Mainly, experts execute physical checkups of skin lesions by examining their appearance, structure, and size. However, such an examination process is a time-consuming activity because of the shortage of dermatologists. The timely detection of melanoma lesions is vital as it can control the mortality rate of victims and also protect them from painful surgical processes. Now, computer vision (CV) and artificial intelligence (AI) approaches have assisted the research community to design valued and computer-aided melanoma diagnostic techniques.

Research areas of AI which are working in the medical area are widely distributed into two types namely the machine learning (ML) and DL frameworks. For the ML techniques, the researchers employ pattern-based methods to extract the feature vector from the input samples which are later classified into respective classes with the help of some classifiers like KNN, SVM, and decision tree (DT). However, due to the complex structural properties of melanoma lesions, the conventional ML approaches are not found much proficient as the extensive changes in the color, size, and shape of lesions decrease the recognition ability of these methods. To enhance the effectiveness of the melanoma recognition systems, classification is performed after segmenting the diseased area of skin from the healthy region. Hence, the segmentation of diseased portions is mandatory for the reliable detection of melanoma lesions. Some works like those performed in [3, 4] have tried to better elaborate the significance of the segmentation procedure. However, the segmentation accuracy of existing approaches have been dropped significantly for images with intense changes in light, brightness, and sample distortions, whereas for practical cases, it is impossible to obtain samples with unchanged characteristics. Therefore, there exists a demand for a more accurate melanoma moles recognition system.

Recently, the robustness and better recall ability of the DL frameworks have encouraged scientists to test them in the area of medical image analysis. These systems have exhibited tremendous performance in several areas of the medical including eye abnormalities recognition [5], brain cancer identification [6], heart diseases [7], and skin cancer classification [8]. The ability of DL methods to better compute ambiguity in health-related systems has empowered them to effectively identify and locate the unhealthy regions of the human body [9–16]. The DL approaches highly rely on the selected convolutional neural network (CNN) that is responsible for extracting useful information from the input images and assisting to locate the diseased portion like melanoma moles from human skin. Several methods have used the CNN frameworks for the timely recognition of the melanoma moles from the dermoscopic images and showed impressive results which clearly depict the better adaptability of these methods toward skin cancer recognition. However, most of these methods accomplished some preprocessing steps to tackle the problem of keypoints maps saturation [17]. To avoid such issues, many works have utilized sample mapping along with pixel-wise labels [18, 19]. However, there is a demand for a more reliable model that can better tackle the issues of existing methods.

Reliable and timely detection of skin cancers from images with intense distortion like the incidence of noise, blurring, and brightness variations is still a complex procedure. Besides, the complex properties of skin moles containing alterations in the size, architecture, and color further enhance the difficulty of the recognition method. Moreover, the presence of hair and small blood vessels also hinders the accurate localization of the diseased region. In this work, we attempted to deal with the problems of existing works by proposing a more robust framework. We have used the CornerNet model along with the FKM approach to detect and segment the skin lesions from the dermoscopic images. The presented work provides the following main contributions:

- Employed CornerNet model with the FKM approach for calculating a reliable set of features, which resulted to improve segmentation ability by locating the moles of varying sizes
- Present a more robust framework that reduces the model train and test time complexity due to its power to tackle the framework overfitted data
- The presented framework is capable of identifying the abnormal skin moles for samples with intense chrominance, brightness, changes, and suffering from noise and blurring attacks
- Enhanced skin lesion segmentation ability of the presented work because of the ability of the CornerNet model to nominate a more representative set of features
- A huge evaluation has been carried out on two standard databases named ISIC-2017 and ISIC-2018 and confirmed the robustness of the introduced work for the melanoma lesions segmentation

We have followed the following structural scheme for the rest of the article: Section 2 explains the work from history that is already performed for skin cancer moles recognition. The presented framework is explained in detail under Section 3 while the model evaluation results are presented in Section 4. Finally, the work is concluded under Section 5.

## 2. Related Work

In this section, an in-depth analysis of already performed work for moles melanoma detection is performed, and the results of all related works are discussed. Extensive work accompanying conventional ML approaches has been carried out by scientists for the automated recognition of skin cancers from dermoscopic samples. One such work was presented in [20] where a segmentation step was performed to locate the area of interest. Secondly, the features from the processed samples were computed by employing the ABCD rule along with the cooccurrence matrix [21]. Finally, the SVM approach was applied to perform the skin moles categorization task. The work proposed in [20] has attained an average accuracy value of 92.10%. Codella et al. [22] introduced a framework by employing the ensembling technique for skin cancer identification. Initially, the U-Net model was used to segment the moles from the input images, after which the features were computed from the segmented areas via using a sparse coding approach and pattern-based methods. Finally, the SVM model was trained on the computed feature to compute the classification score. In the last step, results are averaged to determine the final class label associated with each image. The work discussed in [22] acquired a classification accuracy of 76% and needs results enhancement. Daghrir et al. [23] proposed a hybrid method to classify the skin moles from dermoscopic images. In the first step, the Otsu method along with the Gaussian approach was used to accomplish the preprocessing step over the input images. Then, the features from the processed samples were extracted by using the scale-invariant feature transform (SIFT) and the histogram of oriented gradients methods, while for the classification of the samples, two renowned ML classifiers named SVM and KNN were used. Besides, the work also proposed a CNN model to compute the deep features as well. The final class associated with each sample is determined by employing a majority vote method. This framework [23] presents improved melanoma lesion identification and categorization results; however, the model requires a huge set of samples for efficient training.

Another framework was presented in [24] that accomplished the melanoma lesion segmentation task by employing the superpixel area growth approach. The approach inherited the idea of the Gaussian mixture model (GMM) which effectively distributed the suspected sample into equal-sized portions. The skin moles were then presented by assigning colored labels to all superpixels extracted with the delta metric approach, which are capable of differentiating the various color shades that are difficult to recognize by the naked eye. The method discussed in [24] attained the segmentation results of 86.83%; however, the segmentation accuracy needs more enhancement. Moreover in [25], another conventional ML approach was introduced that employed the concept of the codeword that used the keypoints similarity computation to accomplish the classification task of melanoma moles. In the first step, a highly correlated set of sample keypoints was computed by employing the linear prediction method, after which the SIFT method along with the RGB color spaces was utilized to compute the feature vector. The acquired feature vectors were employed for the SVM training to accomplish the melanoma moles classification job. The work [25] robustly utilizes processing resources; however, the classification results have highly relied on the selected codebook size.

Now, the efficiency of the CNN and DL approaches has gained much popularity which insisted scientists to test them in the area of health and care as well. Several approaches have been presented by the researcher to provide automated solutions for several medical-related applications like assisting the practitioner in radiology or automatically recognizing several types of syndromes including skin cancers. One such technique was proposed in [26], where the author presented a lightweight framework for recognizing melanoma moles that could be deployed on smartphones. This work utilized a DL method named the AlexNet model and trained it over the HAM10000 dataset to classify the melanoma moles. The work discussed in [26] attained the classification accuracy of 84%; however, unable to perform well for lesions of very small sizes. Acosta et al. [27] introduced a DL framework to locate and categorize skin moles into various classes. Initially, the Mask-RCNN model was employed to identify the diseased area from the input image. Next, a CNN framework namely the ResNet152 was applied over the segmented region to compute the features and specify the class associated with the detected part. The samples were recognized as benign or malignant. Zhang et al. [28] also employed a DL approach where the fully convolution network (FCN) technique was employed to segment the diseased areas from the dermoscopic samples. The used model named the FCN employed the VGG model as its base network for calculating the nominative feature set from the input images. Moreover, a small framework was employed to fuse the texton pattern-based pixel keypoints with the deep features. This work [28] shows better melanoma moles classification performance; however, the detection results reduce on images containing moles with arbitrary shapes and similar looks. Another approach was proposed in [29] where a DL model namely FC-DPN was introduced to enhance the melanoma lesions segmentation results. The FC-DPN model employed the FCN approach by replacing the dense blocks with the dual-path network (DPN) blocks to better extract the sample features. The DPNs were then distributed into two subregions namely the DPN-projection and processing units, respectively. The major reason for this distribution was to successfully reemploy the computed keypoints. The work discussed in [29] attains the classification accuracy of 95.14% over the ISBI 2017 dataset,4 however, at the charge of increased model computational complexity. Lei et al. [30] proposed a work to perform the automated detection and classification of skin cancers. The approach utilized the residual framework with the FCN method to differentiate the healthy and diseased areas from dermoscopic samples. After this, the employed framework was utilized to calculate the estimation of all pixels combined iteratively to produce the resultant segmentation mask of the skin moles. The methodology elaborated in [30] has shown an average segmentation accuracy value of 95.78% over the ISBI 2016 repository; however, this approach is computationally expensive. A similar method was introduced in [31] that employed the pixel-wise contribution of samples to locate the melanoma moles from the input samples. Initially, a step was performed to improve the visual appearance of the dermoscopic images. Then, an encoder-decoder framework was used to process the improved images to accomplish the classification task as normal or melanoma-affected. The framework discussed in [31] is efficient to locate the skin moles from the dermoscopic images; however, the model is suffering from an overfitting problem. An object detection-based approach was proposed in [32] to present an automated system for skin mole detection and segmentation. First, annotations were developed to exactly identify the diseased portion from the training samples, on which a DL approach namely the Faster-RCNN was trained. At the test stage, the trained model locates the melanoma-affected region that was further segmented by the fuzzy *K*-means algorithm. The work was capable of locating the small lesions and acquired the average segmentation accuracy of 95.60% over the PH2 repository. Similarly, many other segmentation approaches [33, 34] have shown robust performance for clustering the diseased areas from the input images.

Nawaz et al. [35] presented another framework for the automated identification and classification of the skin model via employing the Faster-RCNN model together with the SVM approach. The work elaborated in [35] is robust to melanoma classification because of its empowerment to tackle the model overfitting data. However, the works discussed in [32, 35] have highly relied on the selection of hyperparameters in the model training phase. Banerjee et al. [36] also presented a framework for melanoma lesion segmentation. The work utilized an object detection model named the YOLO that extracted a feature vector from the dermoscopic images to locate the position of the affected region. After this, the L-type fuzzy approach was employed to accomplish the segmentation task. The work proposed in [36] performs well for the skin cancer moles segmentation task; however, the detection performance degrades for the lesions of very small sizes. Another DL work was elaborated in [37] where a CNN model was designed to accomplish the skin moles classification task. The CNN model consisted of 68 layers along with the classification unit. The model [37] provides a lightweight solution to melanoma classification; however, results are reported for a small database. Khan et al. [38] proposed a computer-aided framework to locate and classify melanoma moles from the input images. The method initially employed an object detection approach named the Mask-RCNN model to locate and segment the skin moles via computing the deep features with the ResNet50 base network. The detected regions were passed to the DenseNet-201 model to understand the structural description of moles which were later categorized by employing the SVM algorithm. The method elaborated in [38] attained the clustering and categorization results of 93.60% and 96.30%, correspondingly, however, at the expense of increased model complexity. Many other researchers have attempted to classify and segment the skin cancer moles [39–45]; however, there is a demand for performance enhancement. Besides, the expense of processing power for such methods is a substantial barrier in medical applications. An analysis of existing work is presented in Table 1.

## 3. Methodology

**Figure 1:**
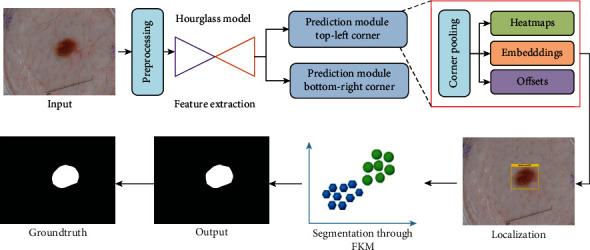
Proposed method diagram.

In the presented work, we have introduced a DL approach namely the CornerNet model along with the FKM method to detect and segment melanoma lesions from dermoscopic images. In the first step, a preprocessing step is executed on the input images to eradicate the unwanted objects from the images under analysis. That is, hair or tiny blood vessels can hinder the recognition ability of the CornerNet model. After this, the processed images are used as input to train the CornerNet model for computing the deep features set and detecting melanoma lesions. Once the moles are detected by the CornerNet model, next the FKM clustering approach is applied to exactly segment the moles. The entire flow of the proposed approach is explained in Figure 1. We have confirmed through analysis that the employment of the CornerNet model with the FKM clustering approach is proficient for locating and segmenting skin lesions of varying masses, shapes, and colors. A detailed description of all steps is given in the proceeding sections.

### 3.1. Preprocessing

The advancement of DL approaches has presented several automated systems for recognizing different medical diseases via employing image modalities. However, the incidence of noise, blurring, and light variation in samples during the capturing process are unavoidable. The major cause of the occurrence of these transformation changes is the quick variations in the lighting conditions and shadows reflected from the human bodies. The presence of such artifacts in samples can reason for performance degradation for any detection model. Furthermore, in the dermoscopic samples of skin cancers, the occurrence of hair and minute blood vessels further increases the complexity of melanoma mole detection and segmentation. To tackle the above-elaborated issues, a preprocessing step is accomplished on the input images by considering several morphological closing operations to remove the unrequired details from images. Besides, an unsharp filter [47] is also applied to enrich the graphic details of the skin cancer samples which contributes to effectively recognizing the melanoma lesions from the dermoscopic images. We have mentioned the mathematical description of the employed preprocessing operation in the following.

(1) $$\begin{array}{c} S_{x}\left(u,v\right)=\left(S\left(u,v\right)⊕W\right)⊖W. \end{array}$$

In Equation (1), *S*(*u*, *v*) is presenting the input sample with *u* and *v* depicting the pixel location. Moreover, *W* presents the structuring kernel with a squared shape along with the size of 10 and angles 90° and 180° for all image values, respectively, while *S*_*x*_(*u*, *v*) denotes processed samples free from artifacts. During the artifacts removal process, some other effects like smoothness and blurring are added to samples, so we have applied an unsharp filter to improve the visual appearance of images. The mathematical construction of the unsharp filter is discussed in the following:

(2) $$\begin{array}{c} S_{p}\left(u,v\right)=S_{x}\left(u,v\right)\times \varpi \left(u,v\right),\\ \varpi \left(u,v\right)=-\frac{1}{\pi \sigma^{4}}\left[1-\frac{u^{2}+v^{2}}{2\sigma^{2}}\;\right]e^{-\left(u^{2}+v^{2}\right)/2\sigma^{2}}. \end{array}$$

Finally, the resultant image *S*_*o*_(*u*, *v*) is attained by employing Equation (3) free from all unnecessary details. After this, the processed images are passed to the CornerNet model for its training to detect the skin moles.

(3) $$\begin{array}{c} S_{o}\left(u,v\right)=S\left(u,v\right)-S_{p}\left(u,v\right). \end{array}$$

### 3.2. CornerNet

The CornerNet [48] is a well-known one-stage object detection model that recognizes the region of interest (RoIs) like the diseased region (skin moles) from the input samples through keypoint calculation. The CornerNet model is concerned to estimate the top-left (TL) and bottom-right (BR) corners to draw the box with more accurateness in comparison to other object detection models [49, 50]. The CornerNet framework is comprised of two basic units which are the feature computation backbone and the prediction module (Figure 1). At the start, a keypoint extractor unit is used which extracts the reliable feature vector that is employed to estimate the heatmaps (Hms), embeddings, offset, and class (C). The Hms is concerned to give the approximation if a specific location in a sample is a TL/BR corner associated with a particular category [51], while the embeddings are used to discriminate the detected pairs of corners and offsets to fine-tune the box position. The corners with high-scored TL and BR coordinates are employed to regulate the exact position of the box, whereas the associated category for each detected diseased region is specified by using the embedding distances on the computed feature vector.

The CornerNet framework shows robust performance in detecting and classifying several types of objects [49, 52–54]. The abnormalities of melanoma lesions have some distinct characteristics, like moles of different shapes and sizes and high color resemblance in the affected and healthy regions of skin areas which complicates the detection procedure. Moreover, the existence of several image distortions like the alterations found in the light, color, and brightness of the samples and the incidence of noise and blurring effect further increase the complexity of the skin lesions detection. Therefore, to better tackle the complexities of samples, we have used the CornerNet model with an Hourglass framework as its base network. The introduced base network is capable of locating and extracting the more relevant sample attributes which assists the CornerNet approach to enhance its recall ability in comparison to the conventional model.

The inspiration for nominating the CornerNet approach for deep features computation and detection of melanoma moles is due to its capability to effectually detect objects by utilizing keypoint approximation in comparison to earlier approaches [49, 50, 55–57]. The framework utilizes detailed keypoints and identifies the object by employing a one-stage detector, so it eliminates the need of using huge anchor boxes for diverse target dimensions than the other one-stage object recognition models, i.e., SSD [55] and YOLO (v2, v3) [56]. Moreover, the CornerNet model is more computationally robust than the other anchor-based two-stage approaches, i.e., RCNN [57], Fast-RCNN [49, 58], and Faster-RCNN [50, 59]) as these techniques employ two phases to accomplish the object localization and categorization job. Consequently, the CornerNet model efficiently tackles the problems of existing works by presenting a more proficient network that extracts more nominative sample features and reduces the computational cost as well.

#### 3.2.1. Hourglass Network

The hourglass network [28] is employed as a backbone to obtain relevant features from the input image. It is a fully convolutional network consisting of one or more hourglass modules. Initially, the model accepts the image with dimensions of 256 × 256. In an hourglass module, input features are first downsampled by using a series of max-pooling layers and convolutions. After that, the features are upsampled back to their original resolution by using a series of convolutional and upsampling layers. As details of the feature are lost during the operation of max-pooling layers, details in the upsampled features are brought back by introducing skip layers. Both global and local features are captured by the hourglass network in one uniform structure. When the network stacks multiple hourglass modules, it can reprocess the features for capturing higher-level information. Due to these properties, the hourglass network becomes a more suitable choice for brain tumor detection.

#### 3.2.2. Corner Detection

After the hourglass network, there are two modules for the prediction of corners, i.e., top-left and bottom-right corners. Each corner has one positive ground-truth position location, and all other locations are set as negative. This is done this way because, for a close pair of false corner detections, a box can still be produced which overlaps the ground-truth box. The radius is determined by analyzing the size of an object, which is done by making sure that a bounding box corresponding to at least an IoU with ground-truth annotations would be generated by a pair of points residing within the radius. The parameter *t* is set to 0.7 in each experiment. When the radius is given, an unnormalized 2D Gaussian is used to determine the amount of penalty reduction. The 2D Gaussian, *g* is determined by *e*^−(*a*^2^ + *b*^2^)/2*σ*^2^^, which has a center at locations and has *σ* as one-third portion of the radius.

The detection loss function is defined as

(4) $$\begin{array}{c} L_{detect}=\frac{-1}{M}\overset{N}{\underset{n=1}{\sum }}\overset{H}{\underset{j=1}{\sum }}\overset{W}{\underset{k=1}{\sum }}\left\{\begin{array}{cc} {\left(1-p_{njk}\right)}^{\varphi }\log\left(p_{njk}\right) & \text{if }g_{njk}=1\\ {\left(1-g_{njk}\right)}^{\omega }{\left(g_{njk}\right)}^{\varphi }\log\left(1-p_{njk}\right) & \text{otherwise}, \end{array}\right. \end{array}$$

where *M* represents the total number of objects in the image. For a certain location (*j*, *k*), *p*_*njk*_ is the score for a certain class C in the set of predicted heatmaps, and *g*_*njk*_ is the heatmap labeled as “ground-truth” which is generated with the Gaussians. *φ* and *ω* are hyperparameters that are responsible for controlling the contribution of each point. In our implementation, we have set the values of *φ* and *ω*, as 3 and 5, respectively. The Gaussian bumps are encoded with *g*_*njk*_, and the term (1 − *g*_*njk*_) guarantees the reduction of the penalty around the locations which are set as ground-truth.

The downsampling layers are employed to reduce memory usage and gather global information [15, 28]. Every location denoted by *a*, *b* in the input image is mapped to another location (*a*/*d*, *b*/*d*) in the heatmaps, where *d* is the factor to which downsampling is performed. Remapping locations from the heatmaps to the input image may result in some precision loss, which can affect the quality of the IoU of smaller bounding boxes. The CornerNet resolves this issue by predicting location offsets for adjusting the corner locations before their mapping to the input resolution and given by

(5) $$\begin{array}{c} Z_{i}=\left(\frac{a_{i}}{n}-\left\lfloor \frac{a_{i}}{n}\right\rfloor ,\frac{b_{i}}{n}-\left\lfloor \frac{b_{i}}{n}\right\rfloor \right), \end{array}$$

where *Z*_*i*_ denotes offset and *a*_*i*_ and *b*_*i*_ are the coordinators of *a* and *b* for corner *i*. Particularly, one group of offsets is predicted shared by top-left corners of all categories, and another group of offsets is shared by bottom-right corners. The smooth L1 loss [11] is applied at ground-truth corners for training purposes and is defined as

(6) $$\begin{array}{c} L_{off}=\frac{1}{M}\overset{M}{\underset{i=1}{\sum }}SmoothL1Loss\left(Z_{i},Z_{i}^{\prime }\;\right). \end{array}$$

#### 3.2.3. Corner Grouping

An image may contain multiple objects; thus, the algorithm may detect multiple bottom-right and top-left corners in a single image. In this step, the algorithm detects the pair of bottom-right and top-left corners belonging to the same bounding box. For each detected corner, the network predicts an embedding vector such that the distance between the embeddings for each bottom-right and top-left corners belonging to the same bounding box should not be large. The corners are then grouped based on these distances. Here, we incorporate the embeddings of only one dimension. The “pull” and “push” losses used to train the network for grouping and separation of the corners are given as below:

(7) $$\begin{array}{c} {Loc}_{pull}=\frac{1}{M}\overset{M}{\underset{i=1}{\sum }}\left[{\left(e_{{tp}_{i}}-e_{i}\right)}^{2}+{\left(e_{{bt}_{i}}-e_{i}\right)}^{2}\right],\\ {Loc}_{push}=\frac{1}{M\left(M-1\right)}\overset{M}{\underset{i=1}{\sum }}\overset{M}{\underset{\begin{array}{c} j=1\\ j\neq i \end{array}}{\sum }}\max\left[0,∆-\left|e_{{tp}_{i}}-e_{j}\right|\right], \end{array}$$

where *e*_*tp*_*i*__  represents the embeddings for the bottom-right corner and top-left corners with *e*_*bt*_*i*__, where *e* denotes embeddings, *tp* denotes top-right corner, *bt* denotes bottom-right corner, and *i* denotes the skin moles. Moreover, *e*_*i*_ denotes the mean of *e*_*tp*_*i*__ and *e*_*bt*_*i*__, and the value of ∆ = 1 is used in all our experiments. Same as the offset loss, the losses are only applied at the ground-truth corner location.

#### 3.2.4. Prediction Module

The feature computation framework has consisted of two separate output units, which denote the TL and the BR corners estimation branches, respectively. Every branch unit comprises a corner pooling layer (CPL) positioned on the top of the backbone to pool keypoints and produces three results: Hms, embeddings, and offsets. This module is an improved residual block (RB) containing two 3 × 3 CnL and one 1 × 1 residual network followed by a CPL. The CPL assists the framework to identify the potential corners. The reduced keypoints are used as input into a 3 × 3 CnL-BtN layer, and then the reverse projection is performed. This improved RB is followed by a 3 × 3 CnL which produces Hms, embeddings, and offsets. The Hms is concerned to give the approximation if a specific location in a sample is a TL/BR corner associated with a particular category, while the embeddings are used to discriminate the detected pairs of corners and offsets to fine-tune the box position. A suspected image can contain more than one affected region; therefore, embeddings assist the model to determine if the predicted corner points belong to a single or different class.

### 3.3. Skin Lesion Segmentation Using FKM

Once the lesions are detected by the CornerNet model, then the FKM approach is applied to the detected moles to segment them by separating the diseased pixels from the healthy regions. The major purpose of nominating the FKM in comparison to the *K*-means clustering technique is that the *K*-means model belongs to the hard clustering category in which one sample belongs to a single cluster. While in comparison, in the FKM approach, one sample can reside in several clusters, so it is better suited to overlapped data.

The detected lesions from the last step are passed as input to the FKM approach to accomplish the segmentation task. The FKM approach distributes the suspected sample into *l* segments *r*_*k*_ = (*k* = 1, 2, 3, ..*l*) that are linked to the center of the cluster denoted as *C*_*l*_. The FKM algorithm employs “fuzzy” or “soft” relation among ROIs and samples and minimizes distortion by utilizing the following formulation:

(8) $$\begin{array}{c} L=\overset{k}{\underset{u=1}{\sum }}\overset{N}{\underset{v=1}{\sum }}b_{u,v}^{f}g_{u,v}. \end{array}$$

In Equation (3), *k* denotes the total clusters, whereas *f* represents the fuzzifier parameter that operates the keypoints and resultant clusters. Moreover, *b*_*u*,*v*_∈ [0,1], and *g*_*u*,*v*_ shows the link and the computed Euclidean distance among the center of clusters and keypoints. A detailed description of the FKM approach is given in Algorithm 1.

## 4. Results

In this part, a detailed description of the used datasets is given. Moreover, we have defined the performance measures used to evaluate the segmentation results of the presented methodology. Furthermore, a comprehensive experimental evaluation has been carried out to explain the segmentation ability of our work in comparison to other latest frameworks.  Table 2 shows the description of the trainable parameters for the proposed approach.

### 4.1. Datasets

**Figure 2:**
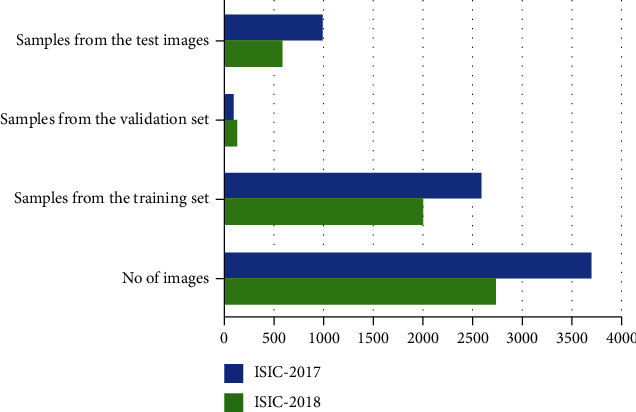
Details of samples from both employed datasets.

To validate the model in segmenting the melanoma moles, we have considered two standard databases named ISIC-2017 and ISIC-2018. The mentioned datasets are provided by the “International Symposium on Biomedical Images (ISBI) in the Challenge of Skin Lesion Analysis toward Melanoma Detection” [60]. A detailed explanation of the employed repositories is elaborated in Figure 2. In both datasets, the ground-truths are provided which are examined and verified by a panel of experts in this domain. The major cause of nominating the ISIC repositories for evaluating the segmentation ability of the presented work is that both datasets contain samples of varying attributes like the intense changes in the size, mass, shape, and color of the moles. Moreover, images are subject to several distortions like containing blurring, noise, and intensity variations which make them challenging and close to real-world examples. We have divided the datasets into three sets where 60% of data is used for model training, 10% for validation, and 30% is employed for model testing.

**Figure 3:**
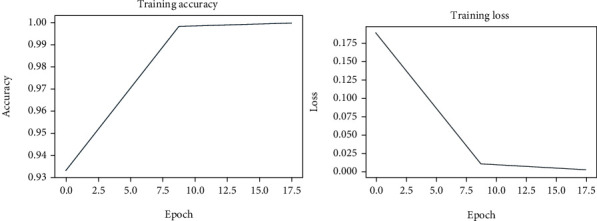
Pictorial illustration of train time accuracy and loss graphs.

We have trained the presented work with an epoch rate of 20. To show the effective learning of our work, we have reported the training accuracy and loss graphs in Figure 3 which are clearly depicting the robust performance of our approach.

### 4.2. Evaluation Metrics

To measure the segmentation results of the presented work, several standard measures named sensitivity [61], specificity [62], accuracy [63], dice coefficient [64], and Jaccard Index [65] are nominated. The mathematical depiction of the used measures is given in the following:

(9) $$\begin{array}{c} \text{Specificity}=\frac{TP}{TP+FP},\\ \text{Sensitivity}=\frac{TP}{TP+FN},\\ Accuracy=\frac{TP+TN}{TP+FP+TN+FN},\\ Dice=\frac{2\times TP}{2\times TP+FN+FP}. \end{array}$$

### 4.3. Assessment of Presented Framework

**Figure 4:**
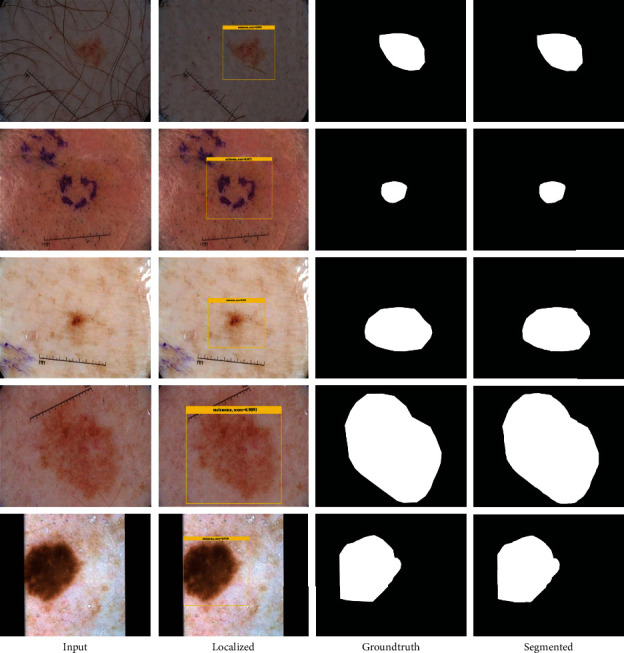
A pictorial representation of both localized and segmented images with the CornerNet model along with the FKM approach.

A precise skin mole detection and segmentation approach should be empowered to accurately locate the lesions of variable mass and structure. To check this, an analysis is performed in this section. For this reason, the test images from both nominated ISIC datasets are taken and evaluated on the trained framework. The test samples from both repositories contain cancer moles of arbitrary shape and size with extensive changes in the chrominance appearance. The visual results for both the detection and segmentation of melanoma moles are shown in Figure 4. The effective recognition ability of the CornerNet model empowers it correctly identify the melanoma lesions by altering mass and structures. To numerically determine the recognition power of the CornerNet model, we employed the mAP performance measure as it helps in understanding the capability of a framework in locating the melanoma moles. The CornerNet model identified the melanoma moles with the mAP value of 0.967, 0.988, and 0.971 over the ISCI-2017 and ISIC-2018 repositories, respectively. For segmentation results, the FKM approach grouped the skin cancer moles with white color to determine the region of interest, while the remaining information is referred to as the black area in the segmented samples. The visual results from both datasets are shown in Figure 4 from where it is quite evident that the proposed approach is proficient in recognizing the melanoma moles and vigorous to variations exist in the position, volume, and architecture of the skin moles.

**Figure 5:**
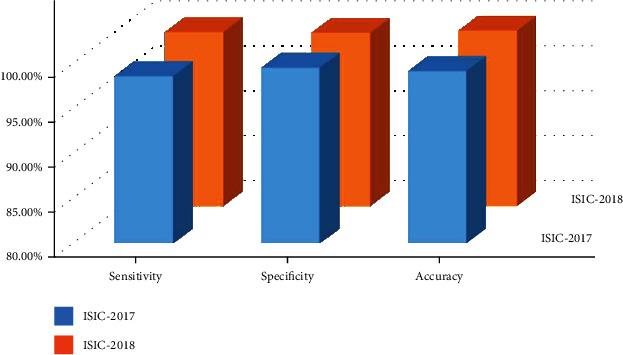
Dataset-wise attained segmentation results.

To further discuss the recognition ability of our method, numerous standard performance metrics are selected to numerically show the robustness of our approach. Initially, we have discussed the sensitivity, specificity, and accuracy values attained over the ISIC-2017 and ISIC-2018 repositories, and the acquired values are shown in Figure 5. Descriptively, over the ISIC-2017 repository, the presented approach reported the sensitivity, specificity, and accuracy values of 98.76%, 99.68%, and 99.32%, respectively, whereas for the ISIC-2018 dataset, the introduced methodology has shown the values of 99.48%, 99.39%, and 99.63% for the sensitivity, specificity, and accuracy measures, respectively.

**Figure 6:**
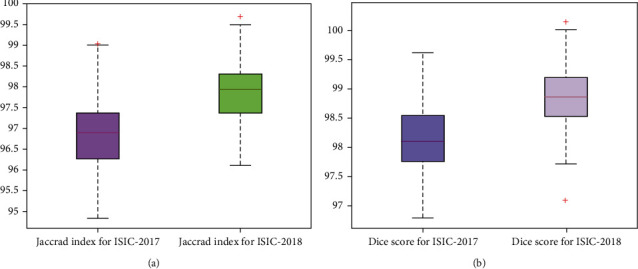
Performance measure of the presented technique in the forms of attained (a) Jaccard index and (b) dice score over the ISCI-2017 and ISIC-2018 datasets correspondingly.

Then, we have selected the Jaccard index and dice coefficient performance metrics as these are considered the standard measures by the researchers for discussing the segmentation results of a model. These measures assist to determine how much a proposed model is capable of locating and recognizing the skin moles of varying sizes and shapes. The attained results are depicted with the help of a box plot as this graph is capable of better discussing the attained values by showing the lowest, highest, and average results (Figure 6). The values shown in Figure 6 clearly show that our work is empowered to depict better segmentation results for both nominated datasets. More clearly, in the case of ISIC-2017 respiratory, the presented technique has attained the Jaccard index and dice scores of 0.9693, and 0.9813, while for the ISIC-2018, the proposed approach has shown the Jaccard index and dice scores of 0.9783 and 0.9886. The values clearly show that our work is quite proficient in locating the lesions under huge changes in the shape and structure of moles.

**Figure 7:**
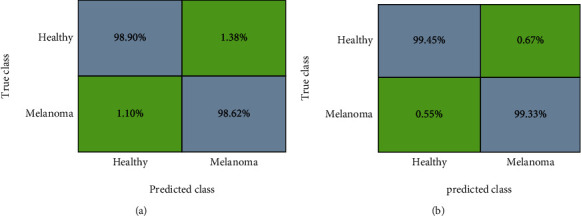
Attained confusion matrix results for the employed databases as (a) ISIC-2017 and (b) ISIC-2018 correspondingly.

Another important measure employed by the scantiest to elaborate on the recognition ability of any model is the confusion matrix as this plot assists to understand the ability of the model to differentiate the healthy samples from the diseased. The attained confusion matrix for both ISIC-2017 and ISIC-2018 repositories with the help of the proposed approach is shown in Figure 7. The reported values in the figure clearly show the effectiveness of our approach in accurately locating and recognizing the diseased and healthy samples.

It is quite evident from the visual and quantitative results discussed above that the proposed framework has exactly segmented the skin moles with a high recall ability and robust performance results. The model has depicted better results because of its power to nominate the representative set of keypoints capable of better discussing the structural information of suspected samples which eventually improves the segmentation results of the proposed approach.

### 4.4. Comparison with Challenge Teams

We performed an analysis to evaluate the segmentation results of the proposed approach for both employed results against the highest performing teams from the ISIC-2017 and ISIC-2018 competitions.

For the ISIC-2017 dataset, the obtained analysis with the challenge teams is shown in Table 3 where we have taken the five highest performing teams and compared our results with them. The stated values in Table 3 are reported from the ISIC-2017 challenge leaderboard. It is quite visible from the comparison shown in Table 3 that the proposed approach has attained the highest segmentation values for all employed evaluation metrics compared to the competitive approaches. The approaches discussed in [66–70] acquired the accuracies of 93.40%, 93.20%, 93.40%, 93.10%, and 93.0%, while comparatively, our work has shown the accuracy results of 99.32%. Similarly, for the Jaccard index, dice score, specificity, and sensitivity, the proposed approach has shown the values of 0.9693, 0.9813, 99.68%, and 98.76%, respectively, which are higher than the values shown in [66–70]. More descriptively, for the Jaccard index metric, we have shown a performance gain of 20.91% which is 13.81% for the dice measure in comparison to the competitive method. Similarly, for the accuracy performance metric, we have attained a performance gain of 6.10%. Besides, for the specificity and sensitivity evaluation measures, we have shown a performance gain of 2.02% and 17.22%, respectively, in comparison to the selected methods. Hence, we can say that our approach is more competent than the peer approaches in segmenting skin lesions and shows the state-of-the-art performance.

Further, we have elaborated on the comparative analysis of our work with the top three teams of the ISIC-2018 challenge to conduct a performance comparison for the ISIC-2018 repository. The obtained comparison is shown in Table 4. For the ISIC-2018 dataset, the proposed approach has performed better than all the selected teams from the competition leaderboard. More clearly, for the Jaccard index, the selected models have shown the average results of 0.8363, which is 0.9783 for our work. Therefore, for the Jaccard index, the proposed work has given an average performance gain of 14.20%. Similarly, for the dice measure, the competitor methods have elaborated the average score of 0.9001, which is 0.9886 for our work. So, we have given an average performance gain of 8.79%% for the dice score. Similarly, for accuracy, the peer teams have shown average results of 94.33%, while our work has gained an accuracy value of 99.63%. So, for the accuracy measure, we have attained an average performance gain of 5.30%. Moreover, for the specificity metric, the nominated teams have attained an average score of 95.96%, which is 99.39% for our method. So, for the specificity measure, we have given a performance gain of 3.42%. Furthermore, for the sensitivity measure, the comparative approaches have given an average value of 91.93%, which is 99.48% for our approach. So, for the sensitivity measure, we achieved a performance gain of 7.55%. In accordance with the conducted evaluation, this analysis demonstrates that the advised method is accomplished in attaining better segmentation results as compared to top-ranked approaches on the same datasets.

### 4.5. Performance Analysis with the Latest Methods

In this part, we have selected numerous new techniques employing the ISIC-2017 and ISIC-2018 datasets and compared our results with them by using several standard performance metrics.

Initially, for the ISIC-2017 datasets, the approaches described in [71–78] are selected, and the attained performance comparative analysis is exhibited in Table 5. Descriptively, the work in [71] was based on a deep learning framework for accomplishing the automated segmentation of the skin moles. In the first phase, the model employed the ResNet50 network to calculate a set of feature vectors, in which later, the segmentation was applied with the decoder unit. This approach [71] exhibited segmentation accuracy, Jaccard index, and dice scores of 94.50%, 80.53%, and 87.92%. Wu et al. [72] proposed a DL framework for recognizing the skin moles and acquired the average accuracy, Jaccard index, and dice scores of 93.26%, 76.53%, and 85.00%, while the work presented in [74] represents a model namely W-net for segmenting the melanoma lesions and reported sensitivity, specificity, and accuracy with the values of 0.9486, 0.9889, and 97.94%. The methods discussed in [73, 75] attained the average segmentation results with accuracies of 98.67% and 86%, respectively. Moreover, the approaches in [76–78] also showed promising segmentation results with values of 94.08%, 94.30%, and 93.13%, respectively, while it is quite evident from the results reported in Table 5 that the proposed approach has shown the highest performance values for all used evaluation measures.

It is quite clear from the values shown in Table 5 that the comparative methods have shown an average sensitivity value of 0.8890, while our work has depicted a sensitivity score of 0.9876 and given a performance gain of 9.86%, while for the specificity measure, the competitive approaches have shown a value of 0.9806 which is 0.9968 for our work. So, we have shown a performance gain of 1.62% for the specificity measure. Moreover, for the accuracy, Jaccard index, and dice score, the selected methods have shown the average values of 99.13%, 81.29%, and 89.37%, which are 99.32%, 96.93%, and 98.13% for the proposed approach. Therefore, we have presented the performance gains of 4.20%, 15.64%, and 8.76% for the accuracy, Jaccard index, and dice score.

We have also performed the comparison of our approach for the ISIC-2018 dataset against the latest approaches mentioned in [72–76, 79–81], and the attained comparison is given in Table 6. In [72], a network named FAT-Net was proposed to perform the segmentation of skin moles from the dermoscopic images and attained sensitivity, specificity, and accuracy scores of 0.9100, 0.9699, and 95.78%, while the work in [74] also utilized a CNN model and depicted the sensitivity, specificity, and accuracy scores of 0.9554, 0.9840, and 97.39%, whereas the method described in [73] deployed a deep learning framework and attained sensitivity, specificity, and accuracy scores of 0.9910, 0.9878, and 98.86%. Araújo et al. [79] presented a method named LinkNet to locate and clustered the melanoma lesions and gained an accuracy score of 96.70%. Furthermore, the approaches elaborated in [75, 81] demonstrate sensitivity scores of 0.86 and 0.7890, respectively. Moreover, the techniques discussed in [76, 80] presented accuracy results of 97.20% and 96.19%, respectively.

The performed analysis in Table 6 clearly shows the effectiveness of our approach in comparison to the latest methods, as we have attained the highest results for all reported performance measures. In a more descriptive manner, the selected approaches have shown an average sensitivity score of 0.8927, while our work has reported a sensitivity value of 0.9939 and given a performance gain of 10.12%. For the specificity, the comparative methods have given a score of 98.14% which is 0.9914 for the proposed work. So, we have shown a performance gain of 1.34% for the specificity measure. Moreover, for the accuracy, Jaccard index, and dice score, the comparative methods have shown average values of 97.02%, 88.78%, and 91.73%, while the proposed approach has demonstrated the values of 99.63%, 97.83%, and 98.86% and presented the performance gains of 2.61%, 9.05%, and 7.13% for the accuracy, Jaccard index, and dice score, respectively.

The performance evaluations conducted in Tables 5 and 6 are clearly confirming the proficiency of our work as compared to the other latest methods. Our approach has gained the highest results due to the shallow structural description of the proposed approach that assists it to avoid the gradient vanishing problem and makes it capable of learning the complex properties of melanoma lesions. The other approaches from history are employing very deep networks with the employment of redundant information which enhances their computational complexity and causes the model overfitting problem. The proposed approach has better tackled the limitations of peer methods by proposing an efficient approach that assists to learn a more descriptive set of the features of the sample and enhances the recall ability of the model.

## 5. Conclusions

The adverse stage of melanoma cancer results in complicated and expensive surgical cure processes and even can cause the demise of the victim. In the presented work, we attempted to diagnose the lesions at the earliest stage by segmenting them from the dermoscopic samples. We have used the CornerNet model along with the FKM approach to detect and segment the skin moles from the dermoscopic samples. Our work is capable of locating and segmenting skin lesions of varying mass, orientations, and colors. Moreover, the proposed approach can easily tackle the incidence of noise, blurring, and intensity changes found in the input images. We have performed a rigorous experimental evaluation over two standard repositories named the ISIC-2017 and ISIC-2018 and attained accuracy scores of 99.32% and 99.63%. Moreover, we have shown the visual samples to elaborate on the accurate segmentation performance of our approach and confirmed that the proposed solution is proficient in accurately recognizing the skin moles and assists the dermatologist in quickly detecting the lesions to understand the severity level of the disease. The proposed framework shows enhanced melanoma lesions detection and classification results; however, small performance degradation is observed for images with extensive color variations. Therefore, in the future, we plan to investigate other DL frameworks along with feature selection techniques to deal with this limitation [82]. Moreover, we planned to evaluate the presented work on other medical diseases.

## Figures and Tables

**Algorithm 1:**
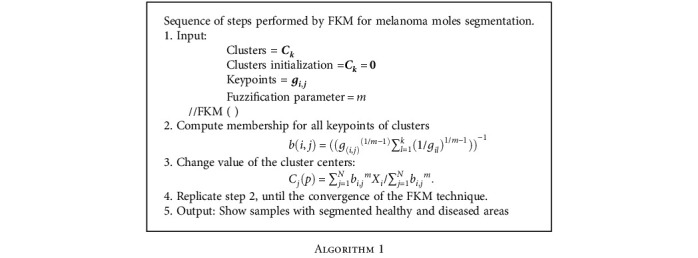


**Table 1:** Relative investigation of existing approaches for recognizing melanoma moles.

Author	Year	Approach	Task	Database	Accuracy
*ML techniques*					
Alquran et al. [20]	2017	Pattern features + SVM	Categorization	Custom database	92.10%
Codella et al. [22]	2017	U-Net + SVM	Categorization	ISIC-2016	76%
Daghrir et al. [23]	2020	SIFT + SVM and KNN	Categorization	ISIC-2017	88.40%
Bama et al. [24]	2021	GMM model	Segmentation	PH2	86.83%
Hu et al. [25]	2019	SIFT + SVM	Categorization	PH2	82%
Durgarao et al. [44]	2021	LVP, and LBP + *C*-means	Segmentation	PH2	79.44%
*DL techniques*					
Ameri et al. [26]	2020	AlexNet	Categorization	HAM10000	84%
Acosta et al. [27]	2021	ResNet-152	Categorization	ISIC-2017	90.40%
Zhang et al. [28]	2019	VGG-16	Categorization	ISIC-2017	92.72%
Shan et al. [29]	2020	FC-DPN	Segmentation	ISIC-2017	95.14%
Bi et al. [30]	2019	Res-FCN	Segmentation	ISIC-2016	95.78%
Adegun et al. [31]	2019	Encoder-decoder	Categorization	ISIC-2017	95%
Nawaz et al. [32]	2021	Faster-RCNN + FKM	Segmentation	PH2	95.6%
Nawaz et al. [35]	2021	Faster-RCNN + SVM	Categorization	ISIC-2016	89.10%
Banerjee et al. [36]	2020	YOLO + L-type fuzzy clustering	Segmentation	ISIC-2017	97.33%
Iqbal et al. [37]	2021	CNN	Categorization	ISIC-2019	88.75%
Khan et al. [38]	2021	Mask-RCNN, DenseNet201 + SVM	Segmentation	ISIC-2016	93.6%
Mohakud et al. [39]	2022	Encoder-decoder	Segmentation	ISIC-2016	98.32%
Abdar et al. [40]	2021	Bayesian model	Categorization	Kaggle skin cancer dataset	88.95%
Pacheco et al. [41]	2021	Metadata and block-based method	Categorization	ISIC-2019	74.90%
Wang et al. [42]	2022	U-Net	Segmentation	ISIC-2017	94.67%
Zhao et al. [43]	2022	U-Net++	Segmentation	ISIC-2018	95.30%
Ali et al. [46]	2021	DCNN	Categorization	HAM10000	91.93%

**Table 2:** Training parameters of the presented methodology.

Model parameters	Value
No of epochs	20
Value of learning rate	0.0001
Selected batch size	8
The threshold for the confidence score	0.2
The threshold for the unmatched region	0.5

**Table 3:** Comparative analysis of our work with performance values from the ISIC-2017 challenge leaderboard.

Method	Jaccard index	Dice	Accuracy	Specificity	Sensitivity
CDNN [66]	0.7650	0.8490	93.40%	97.50%	82.50%
U-Net [67]	0.7620	0.8470	93.20%	97.80%	82.00%
Deep residual network [68]	0.7600	0.8440	93.40%	98.50%	80.20%
U-Net [69]	0.7540	0.8390	93.10%	96.90%	81.70%
FCNN [70]	0.7.20	0.8370	93.00%	97.60%	81.30%
Proposed	0.9693	0.9813	99.32%	99.68%	98.76%

**Table 4:** Comparative analysis of our work with performance scores from the ISIC-2018 challenge leaderboard.

Method	Jaccard index	Dice	Accuracy	Specificity	Sensitivity
Mask-RCNN2+segmentation	0.838	0.898	94.20%	96.30%	90.60%
Ensemble with CRFv3	0.837	0.904	94.50%	95.20%	93.40%
Lesion segmentation by DCNN	0.834	0.900	94.30%	96.40%	91.80%
Proposed	0.9783	0.9886	**99.63%**	99.39%	99.48%

**Table 5:** Performance analysis of the proposed work with the new methods over the ISIC-2017 dataset.

Reference	Sensitivity	Specificity	Accuracy (%)	Jaccard index (%)	Dice (%)
[71]	0.8804	0.9659	94.50	80.53	87.92
[72]	0.8392	0.9725	93.26	76.53	85.00
[74]	0.9486	0.9889	97.94	—	93.22
[73]	0.9695	0.9950	98.67	95.98	97.95
[75]	0.8600	—	—	—	—
[76]	—	—	94.08	78.55	86.48
[77]	—	—	94.30	—	—
[78]	0.8364	—	93.13	74.88	85.63
Proposed	0.**9876**	0.**9968**	**99.32**	**96.93**	**98.13**

**Table 6:** Performance analysis of the proposed work with the new methods over the ISIC-2018 dataset.

Reference	Sensitivity	Specificity	Accuracy (%)	Jaccard index (%)	Dice (%)
[72]	0.9100	0.9699	95.78	82.02	89.00
[74]	0.9554	0.9840	97.39	—	93.00
[73]	0.9910	0.9878	98.86	95.66	97.78
[79]	0.8620	0.9860	96.70	80.00	88.90
[75]	0.8600	—	—	—	—
[80]	0.8691	0.9809	97.20	97.57	—
[76]	0.9049	—	96.19	83.45	89.99
[81]	0.7890	0.9800	—	94.00	—
Proposed	0.**9939**	**0.9948**	**99.63**	**97.83**	**98.86**

## Data Availability

Two publically available datasets have been utilized for the experimental process such as ISIC-2017 and ISIC-2018 (https://challenge.isic-archive.com/challenges/).
